# Clinical Features, Diagnosis, and Treatment of Primary Intraventricular Lymphoma: Insights From a Monocentric Case Series

**DOI:** 10.3389/fneur.2022.920505

**Published:** 2022-06-06

**Authors:** Lidong Cheng, Hongtao Zhu, Jing Wang, Guanghui Wang, Xiaoyu Ma, Kai Zhao, Junwen Wang, Kai Shu

**Affiliations:** Department of Neurosurgery, Tongji Hospital, Tongji Medical College, Huazhong University of Science and Technology, Wuhan, China

**Keywords:** primary central nervous system lymphoma, intraventricular, clinical features, diagnosis, treatment, case reports, review

## Abstract

**Objective:**

Primary ventricular lymphoma (PVL) is an extremely rare and commonly misdiagnosed disease. Previous studies were predominantly case reports, and literature regarding the diagnosis and treatment of PVL is limited. Therefore, this study aimed to evaluate the characteristics of patients with PVL.

**Methods:**

The data of patients with pathologically confirmed PVL were assessed. Epidemiological data, imaging findings, surgery, pathological results, and prognosis were retrospectively analyzed. A systematic review of relevant literature was also conducted.

**Results:**

A total of eight patients with PVL were identified. The main symptom was increased intracranial pressure. Radiographically, five patients had single lesion and three had multiple lesions; typical findings on magnetic resonance imaging included hypointensity on T1- and T2-weighted imaging, adjacent brain edema, and homogeneous enhancement on contrast-enhanced T1-weighted images. Preoperatively, six cases were misdiagnosed and two cases did not get a definite diagnosis. Craniotomy was performed on all patients, and four achieved gross total resection. Hydrocephalus was relieved after surgical resection in four patients. Pathology revealed diffuse large B-cell lymphoma in all patients. Only one patient had a severe complication. A total of three patients received concomitant adjuvant treatment, whereas five patients refused any adjuvant therapy. At the time of follow-up, the median survival time of patients was 15 months.

**Conclusion:**

Primary ventricular lymphoma mainly presented with symptoms of increased intracranial pressure and had several imaging characteristics for the diagnosis, but the condition still tends to be misdiagnosed. Surgical resection is a feasible treatment for patients with isolated nodules, especially those with acute obstructive hydrocephalus.

## Introduction

Primary central nervous system lymphoma (PCNSL) refers to lymphoma that grows solely within the brain, spinal cord, and eyes, without systemic involvement. PCNSL is a relatively rare and highly invasive extra-nodal non-Hodgkin's lymphoma, accounting for ~2–5% of intracranial tumors and 4–6% of extra-nodal lymphomas (1, 2). The disease is common in patients with immunodeficiency, but recent literature has reported an increasing incidence of PCNSL in populations with normal immunity (3). PCNSL is associated with poor clinical outcomes and has a median progression-free survival time of 12 months and a median overall survival time of ~3 years (4). More than 90% of PCNSLs are located in the brain parenchyma of the cerebral hemispheres, whereas primary lymphomas in the ventricle are extremely rare. Notably, there is a paucity of information on the diagnosis, treatment experience, and patient characteristics in PCNSL. In this study, we report the on cases of primary ventricular lymphoma (PVL) at our center and summarize the clinical characteristics and therapeutic effects of PVL based on a literature review.

## Methods

### Study Patients

We performed a thorough review and analysis of the clinical data of patients with PCNSL admitted to our center from January 2010 to December 2020. The inclusion criteria were pathological findings confirming lymphoma, lesion located solely in the intraventricular system, and normal immune function. Patients with other systemic lymphomas or brain parenchymal lymphoma invading the cerebral ventricular system were excluded. A total of eight of 198 patients with PCNSL were included in this study, and their epidemiological data, imaging findings, surgical conditions, and pathological results were analyzed. A follow-up was conducted to evaluate patient's prognosis. This study was approved by the Ethics Committee of Tongji Hospital affiliated with Tongji Medical College of Huazhong University of Science and Technology. Because of the retrospective nature of the study, patient consent was not required.

### Literature Search

**Figure 1:**
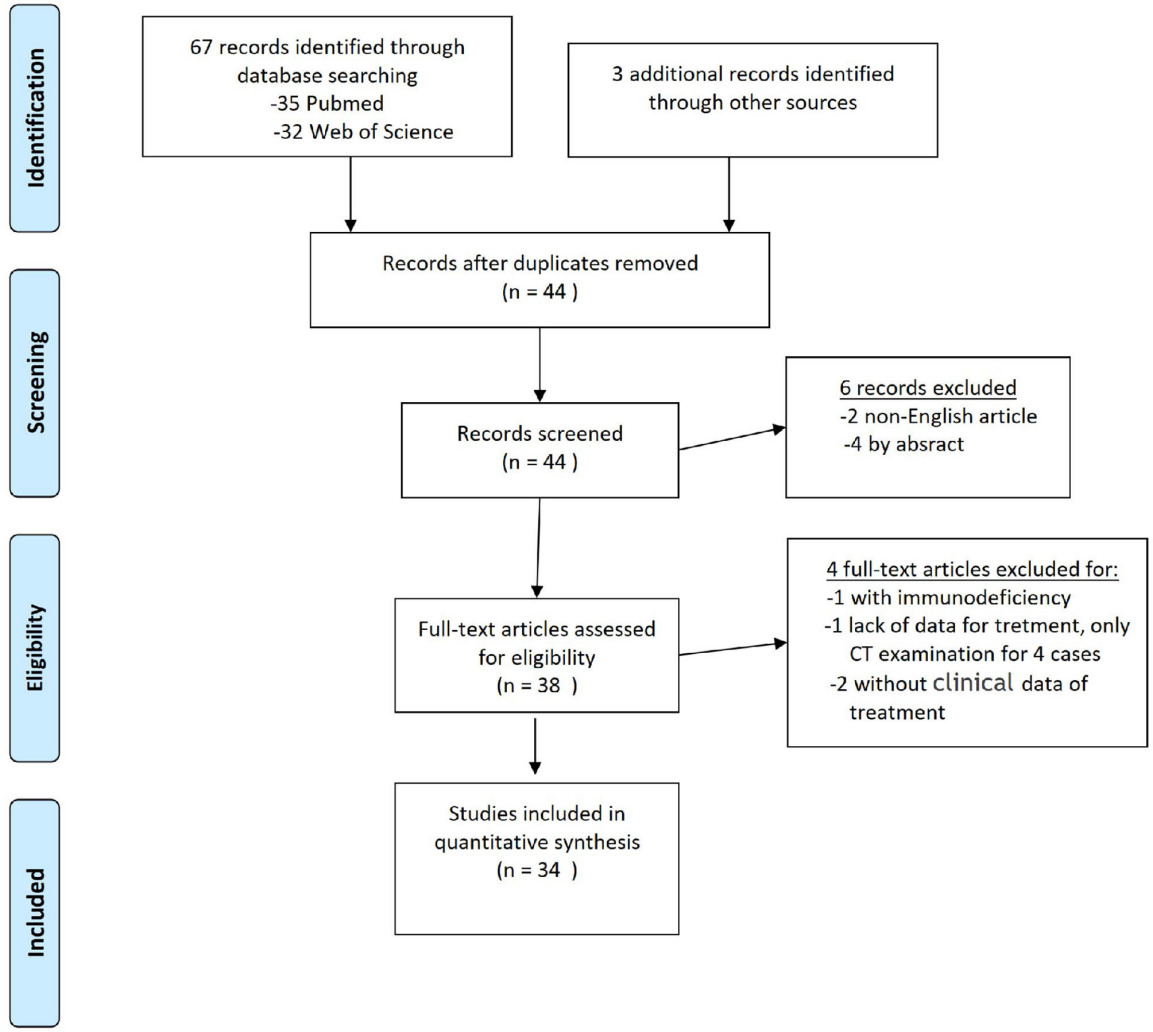
PRISMA flow diagram for literature search. From Moher et al. (5).

A thorough literature screening of the PubMed and Web of Science databases for case reports on PCNSL in the ventricle was conducted according to the guidelines of the Preferred Reporting Items for Systematic Reviews and Meta-Analyses (PRISMA) using the following terms: “lymphoma” or “PCNSL” and “ventricle,” “lateral ventricle,” “third ventricle,” or “fourth ventricle.” The references of the reports were also reviewed. In total, 38 relevant articles were analyzed, 46 patients with a diagnosis were identified, 6 lacked sufficient data, and 1 patient had immunodeficiency; finally, 34 studies for qualitative synthesis were included (Figure 1).

## Results

### Case Series

Epidemiological data, imaging findings, surgery, pathological results, radiotherapy and chemotherapy outcomes, and postoperative survival times of patients are presented in Table 1. Of the eight patients, two were women and six were men, and their age ranged from 35 to 69 years (mean, 55±13 years). The most common symptoms included increased intracranial pressure and impaired cerebellar balance perception. A total of five patients experienced headache and dizziness, three presented with nausea and vomiting, two exhibited walking instability, and one presented with memory loss. The onset of disease varied from 1 week to 2 months.

**Table 1:** Characteristics of the 8 patients included in the series.

**Case no**.	**Age (yrs), sex**	**Presenting** **signs**	**Location**	**Size (cm)**	**HD**	**Growth pattern**	**Extent of resection**	**Complication**	**Adjuvant therapy**	**FU** **(mos),** **status**
1	69, F	Headache, dizziness	4th V	4.0	Y	Solitary nodular	GTR	Lung infection	ND	15, died
2	35, M	Dizziness, vomiting	LV, 4th V	7.0	Y	Cluster like	PTR	N	ND	2, died
3	52, M	Unsteady gait	4th V	3.0	N	Solitary nodular	STR	N	CMT+RT	36, alive
4	68, M	Dizziness, vomiting	4th V	3.1	Y	Solitary nodular	GTR	N	CMT+RT	18, died
5	39, M	Headache	LV	3.0	N	Solitary nodular	GTR	Subdural hematoma	CMT+RT	48, alive
6	64, M	Decline in memory,	LV	3.2	N	Cluster like	PR	N	ND	4, died
7	52, M	Dizziness, unsteady gait, vomiting	4th V	3.1	Y	Solitary nodular	GTR	Respiratory failure	ND	Give up treatment
8	67, M	Unsteady gait	LV, 4th V	1.2	N	Diffuse type	PTR	N	ND	1, died

*M, male; F, female; 4th V, fourth ventricle; LV, lateral ventricle; HD, hydrocephalus; Y, yes; N, no; GTR, gross total resection; STR, subtotal resection; PTR, partial resection; CMT, chemotherapy; RT, radiotherapy; ND, not did; FU, follow-up*.

Preoperative magnetic resonance imaging (MRI) examination revealed single lesions in five cases and multiple lesions in three cases (37.5%). A total of four cases were confined to the fourth ventricle, two cases exhibited involvement of the lateral ventricle, and two cases presented with simultaneous involvement of the lateral and fourth ventricles (patients 2 and 8). The maximum diameter of lesions was 1.8–7.0 cm (average, 3.5 cm). A total of four patients exhibited varying degrees of hydrocephalus. There are several radiological features; typical MRI revealed hypointensity on T1- and T2-weighted imaging, with adjacent brain edema, and homogeneous enhancement on contrast-enhanced T1-weighted images, with cluster-like lesion in two patients (patients 2 and 6) and diffuse growth along the choroid plexus and ventricular wall in one patient (patient 8). Preoperatively, the suspected radiological diagnosis of four patients was medulloblastoma or ependymoma, the radiological diagnosis of two patients was meningioma (patients 5 and 6), and the other two patients did not have a definitive diagnosis.

Craniotomy was performed on all eight patients: 4 (50%) achieved gross total resection (GTR), 1 (12.5%) achieved subtotal resection (STR), and 3 (37.5%) achieved partial resection (PTR). Only one patient experienced complications with pulmonary infection, and one presented with minimal subdural hematoma; both patients recovered after treatment. The most serious complication was observed in patient 7, who presented with respiratory failure after surgery. Postoperatively, this patient received tube incision and respiratory support treatment; however, the patient died 10 days later after his family opted to discontinue treatment. None of the other patients presented with serious complications, and all patients recovered well after the surgery. Patient 3 underwent postoperative protocol chemotherapy with high-dose methotrexate (HDX)+temozolomide in addition to locally enhanced radiotherapy in the whole cerebellum. Patients 4 and 5 underwent HDX regimen chemotherapy and whole-brain radiotherapy. Other patients refused chemotherapy and radiotherapy after the surgery.

Postoperative pathology revealed diffuse large B-cell lymphoma (DLBCL), with immunohistochemically related positive indicators, including CD20, CD79a, LCA, MUM-1, PAX5, C-MYC, BCL-2, and BCL-6, in all patients. Only CD20 in patient 5 was negative. The Ki-67 marker index was 70–100%, with an average of 88%. Molecular pathology was performed in five patients, and EBV-encoded small RNA chromogenic *in situ* hybridization was negative in five patients (Table 2).

**Table 2:** Pathological features of 8 patients.

**Case no**.	**CD20**	**LCA**	**CD79a**	**PAX5**	**MUM-1**	**BCL-2**	**BCL-6**	**C-MYC**	**Ki67(%)**	**EBER CISH**
1	+	+	ND	ND	+	ND	+	ND	80	ND
2	+	+	+	+	–	+	+	+	95	–
3	+	+	+	–	+	ND	+	+	90	ND
4	+	ND	+	+	–	+	+	ND	90	ND
5	_	ND	+	+	–	–	+	+	100	–
6	+	ND	ND	+	+	+	–	+	95	–
7	+	ND	ND	+	+	+	+	–	70	–
8	+	ND	ND	+	+	+	+	+	90	–

*EBER CISH, EBV-encoded small RNAs chromogenic in situ hybridization*.

With the exception of patient 7, who discontinued treatment 10 days after surgery, all other patients recovered and were discharged from the hospital. The survival time of patients ranged from 1 month to 4 years. Patients 3 and 5 were still alive during the reporting of this study. At the time of follow-up, the median survival time of patients was 15 months.

### Combined Cases and Systematic Review

**Figure 2:**
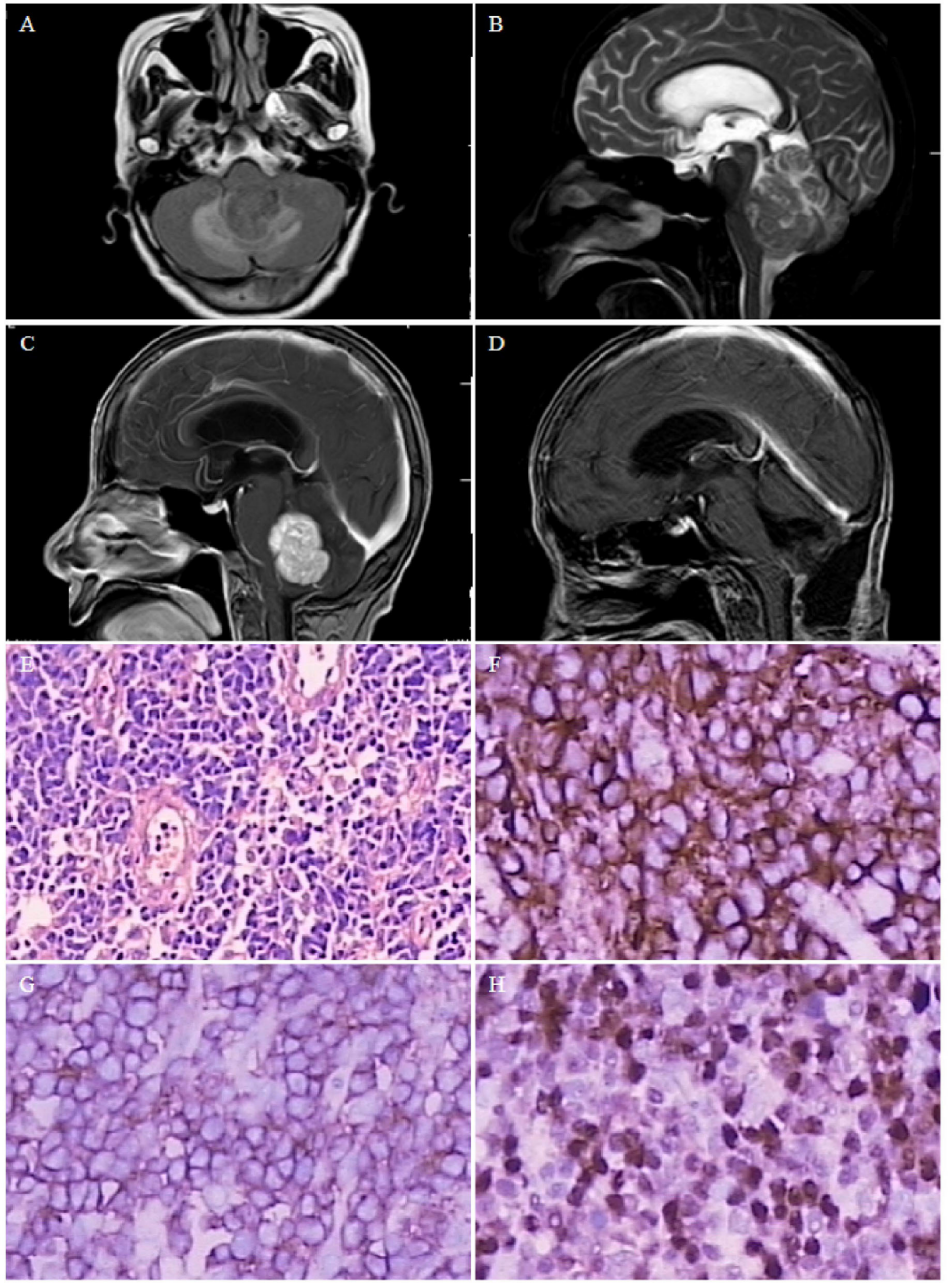
Case 1. Magnetic resonance image of a 4.0×2.8-cm solid mass in the fourth ventricle, with supratentorial hydrocephalus. **(A)** T1-weighted image showing low signal. **(B)** T2-weighted image showing low signal. **(C)** Homogeneous enhancement on contrast-enhanced T1-weighted images. **(D)** Postoperative magnetic resonance image showing no residual lesion, and obstructive hydrocephalus is relieved. Pathological microscopic examination shows diffuse large B-cell infiltration [**(E)** hematoxylin and eosin, 200× magnification], including CD20- [**(F)**, 400× magnification], LCA- [**(G)**, 400× magnification], and MUM-positive [**(H)**, 400× magnification] lesions.

**Figure 3:**
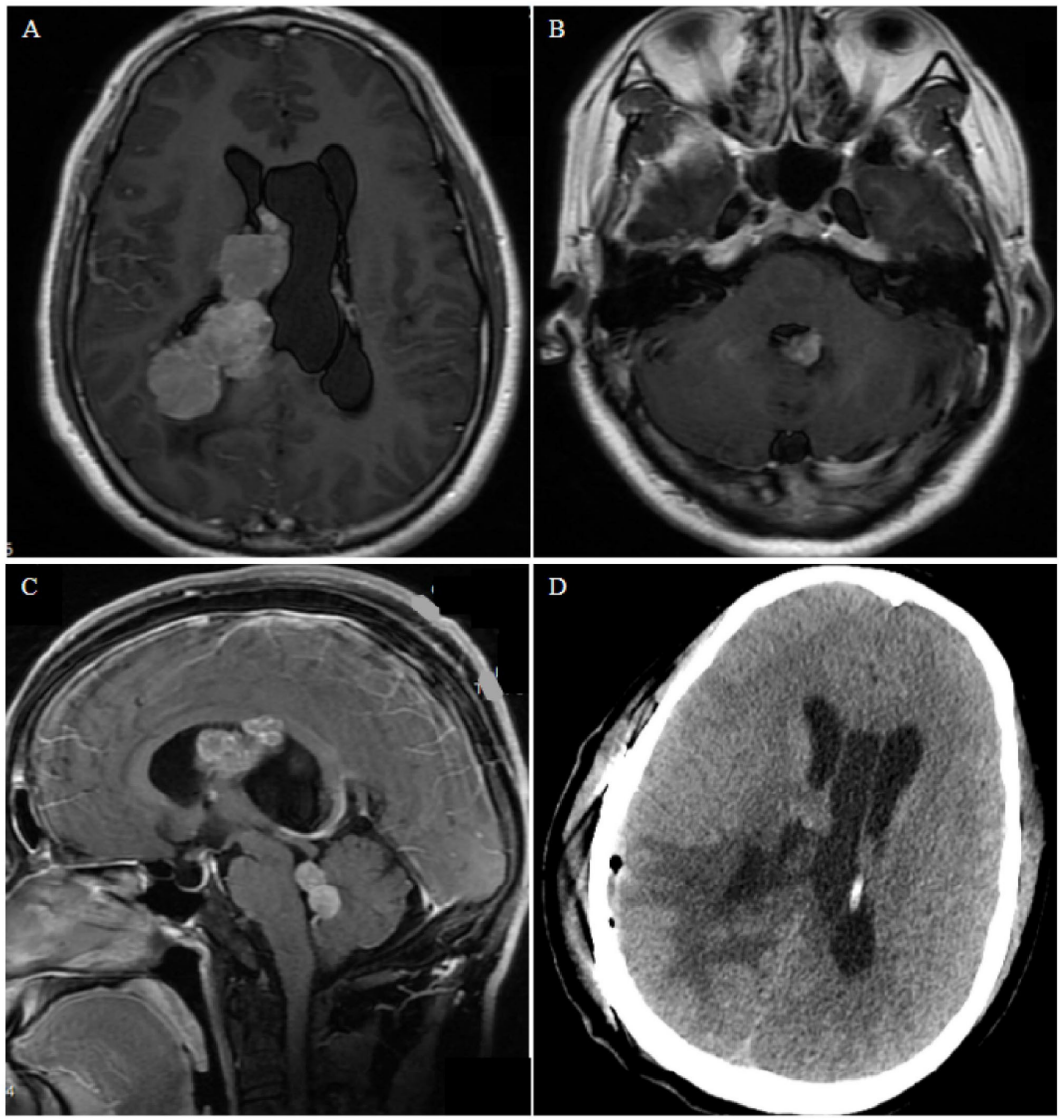
Case 2. **(A,B)** Contrast-enhanced T1-weighted axial images show nodular bead-like lesions in the right and fourth ventricles. **(C)** Sagittal magnetic resonance images showing multiple lesions occupying the midbrain aqueduct and fourth ventricle. **(D)** Postoperative computed tomography shows edema of brain tissue and no bleeding in the operative cavity.

**Figure 4:**
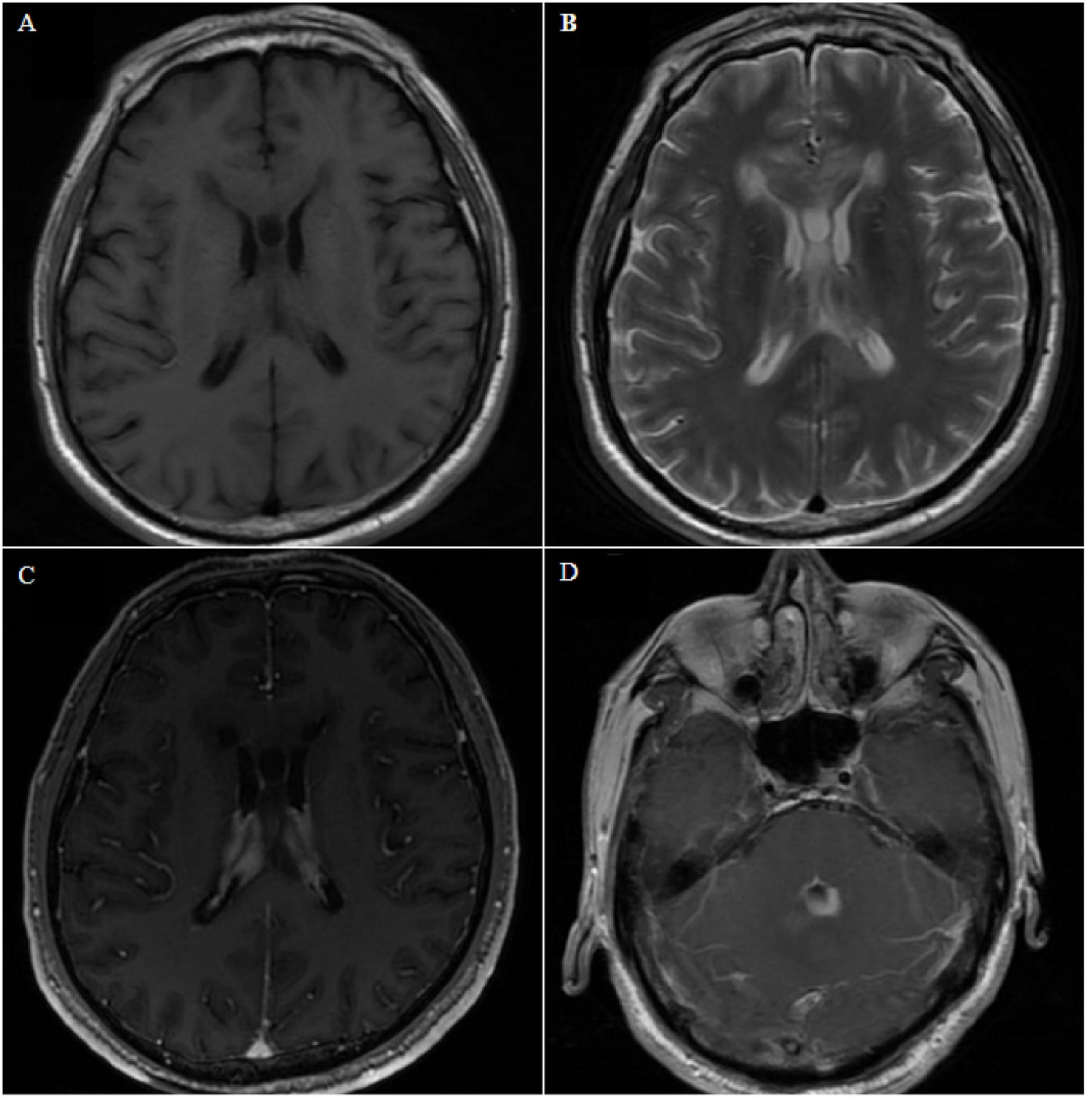
Case 8. Axial magnetic resonance image showing bilateral and fourth ventricle lesions, diffuse growth along the choroid plexus and ventricle wall, low signal on T1- and T2-weighted images **(A,B)**, and homogeneous enhancement on contrast-enhanced T1-weighted images **(C,D)**.

A total of thirty-nine patients were included in this study through a systematic review (Supplementary Table 1) (6–39). In total, 47 patients with PCNSL in the ventricle were identified, including in our series and previous articles. Of all patients, 27.7% were women, and the mean age was 55 ± 18 years (Supplementary Table 2). The most common symptoms were headache, nausea, and vomiting (28/47, 59.6%). Moreover, 25.5% patients presented with hemiplegia, ataxia, gait instability, and other motor disorders; 23.4% presented with dizziness or vertigo; 6 (12.8%) exhibited diplopia; 6 presented with memory deficits; 8 presented with rare symptoms, such as seizures, speech disturbance, and confusion. Among the patients, 59.6% had single lesions and 40.4% had multiple lesions. Lesions were confined to the lateral ventricle in 9 (19.1%) patients, third ventricle in 7 (14.9%) patients, and fourth ventricle in 17 (36.2%) patients. Lesions involving multiple ventricles were observed in 14 (29.8%) patients (including one with intraspinal metastasis). Solitary nodular growth was the most common growth pattern (25/47, 53.2%) (Figure 2). Multiple nodules and cluster-like growth patterns (Figure 3) were observed in 5 (10.6%) and 4 (8.5%) patients, respectively. Diffuse growth along the choroid plexus and ventricular wall was also common (21.3%, 10/47) (Figure 4). A total of fifteen (31.9%) patients had hydrocephalus, of whom 7 had acute hydrocephalus. According to radiological data, 38 (80.9%) patients were misdiagnosed and 9 (19.1%) cases did not get a definite diagnosis.

A total of seven (14.9%) patients underwent urgent external ventricular drain for acute hydrocephalus, and 26 (55.3%) underwent surgical resection, of whom half (50%) achieved GTR and the remaining half (50%) achieved STR or PTR; 40.4% (19/47) were confirmed by biopsy, 2.1% (1/47) was confirmed using cerebrospinal fluid (CSF) cytopathology, and 2.1% (1/47) was confirmed based on postmortem autopsy. According to pathology, BCL was the most common (74.5%, 35/47), followed by Burkitt lymphoma (8.5%, 4/47), T-cell lymphoma (6.4%, 3/47), and small lymphocytic lymphoma (4.3%, 2/47). A total of three patients were pathologically unclassified. After diagnosis, 29.8% (14/47) of the patients received chemotherapy only, 6.4% (3/47) received radiotherapy only, 27.7% (13/47) received concomitant radiotherapy and chemotherapy, 2.1% (1/47) received immunotherapy, and 34.0% (16/47) did not receive any adjuvant therapy.

At the time of reporting, 25.5% (12/47) of the patients had died, with a median survival time of 7.0 ± 7.2 months (range, 1–18 months), and 59.6% (28/47) of the patients were still alive, with a follow-up time of 12.4 ± 10.3 months (range, 0.5–48 months). The prognosis of 7 patients was not reported.

### Case Examples

#### Case 1

A 69-year-old woman presented with headache and dizziness for 2 months, and weakness in both legs 1 week before admission. The patient had previously developed ocular fundus pigmentation, resulting in blindness in both eyes. No immune deficiency or other underlying diseases were noted. The patient underwent head imaging 4 months before admission, which only indicated white matter thinning. Physical examination revealed no obvious positive signs. Brain MRI revealed a 4.0×2.8-cm solid mass in the fourth ventricle (Figure 2), which was considered a medulloblastoma or solid hemangioblastoma. The patient and her family opted for surgical resection. The surgery was performed *via* a prone postero-medial approach. During the operation, the tumor was observed to originate from the lateral orificium choroid plexus of the fourth ventricle with an abundant blood supply. Complete tumor resection was achieved under electrophysiological monitoring. Histopathological examination revealed diffuse large B-cell infiltration (Figure 2E), with positive immunochemical staining for CD20, LCA, and MUM (Figures 2F–H, respectively). The diagnosis was primary DLBCL of the fourth ventricle. The patient developed complications of pulmonary infection, recovered, and was discharged after 2 months of antibiotic treatment. The patient refused further chemoradiotherapy and died 15 months after surgery due to tumor recurrence.

#### Case 2

A 35-year-old man presented with dizziness, nausea, and vomiting for 2 months. No obvious abnormalities were noted on physical examination, and no past underlying diseases were identified. Magnetic resonance imaging (MRI) examination revealed multiple lesions in the right lateral ventricle, middle cerebral aqueduct, and fourth ventricle, some of which were cluster-like, accompanied by hydrocephalus, with avid enhancement (Figure 3). Preoperative diagnosis could not be performed, considering the possibility of metastases. Most of the intraventricular tumors were resected *via* the temporo-occipital approach. During the operation, the lesions were observed to originate from the choroid plexus. Microsurgical resection was uneventful, and postoperative pathology revealed diffuse large BCL, with no significant postoperative complications. The patient refused further radiotherapy and chemotherapy and died 2 months later.

#### Case 8

A 67-year-old man presented with gait instability for 20 days, and he had no history of underlying diseases. MRI examination revealed lesions in bilateral ventricles and the fourth ventricle (Figure 4), which could not be diagnosed clearly before surgery. After microsurgical resection, the lateral ventricular lesion was partially resected. Postoperative pathology revealed diffuse large BCL. Postoperative systemic examination revealed no other systemic lymphoma. The family refused further chemotherapy, and the patient died 1 month later.

## Discussion

Primary central nervous system lymphoma is predominantly located in the deep periventricular parenchyma, such as the corpus callosum and basal ganglia. Lesions may be single or multiple (~34%) (40), and some lesions may extend into the ventricles. Up to 40–100% of parenchymatous subependymal lesions extend to ependymal surfaces (11). However, PCNSL with lesions purely located in the ventricular system is extremely rare. Previous studies were predominantly the case reports. Indeed, we only identified 39 patients with complete information. This study aimed to improve our current understanding of the diagnosis and treatment of PVL, and to this end, we summarized the epidemiological data, clinical manifestations, and imaging characteristics of PVL in detail based on our cohort and previous literature.

### Epidemiology

The average diagnostic age of PCNSL was 65 years, and the male-to-female ratio was ~1.2:1.7. Studies in the last 10 years have reported that the incidence of PCNSL is higher in elderly patients, especially in the population aged 70–79 years (41). In this study, the average age of patients with PVL was 54 years, which was similar to the overall age of PCNSL onset. Most cases were adults (91.5%, 43/47), and only 4 cases were children (<18 years). The male-to-female ratio was ~2.6 (34:13), and PVL was more common in men.

### Presenting Signs

Clinical symptoms of cerebral parenchymal lymphoma varied, with 70% presenting with focal neurologic deficits and 32–43% presenting with mental and behavioral changes; meanwhile, symptoms of increased intracranial pressure were less common than other intracranial tumors (~32–33%), and epilepsy was less common (~11%) (4, 42). PVL typically presented with headache, nausea, vomiting, and other symptoms of increased intracranial pressure, which comprised its main clinical features. Increased intracranial pressure was the reason for medical visits in up to 59.6% of patients. In addition to the mass effect caused by the lesion, intraventricular lymphoma readily blocked the CSF circulation pathway, resulting in hydrocephalus and aggravation of increased intracranial pressure. In our group, four of the eight patients presented with various degrees of hydrocephalus. Focal symptoms caused by lesion compression of adjacent nerves or brain tissue were relatively rare in PVL, accounting for 25.5% of patients. For example, lateral ventricular tumor compression of the thalamus resulted in limb hemiplegia; fourth ventricular tumor compression of the cerebellum induced balance abnormalities, resulting in gait imbalance or ataxia. Other common symptoms included dizziness or vertigo, diplopia, and memory loss. Epilepsy was rare in patients with this disease, with only 2 (4.2%) patients identified. Disturbance of consciousness was observed in a few patients with acute intracranial hypertension or lesions severely compressing the brainstem. Collectively, our findings indicate that increased intracranial pressure is the main clinical symptom of PVL, but this is often nonspecific.

### Imaging Features

More than half of patients with AIDS have multiple lesions, whereas in ~25% of immunocompetent patients, single lesions are more common (43). Of all patients with PCNSL, 87% were supratentorial, and a single lymphoma located in the infratentorial area was extremely rare in patients with normal immune function. PVL with solitary nodules was common, but the proportion of cases with multiple lesions was also high (40.4%). This may be associated with the spread of tumor cells *via* CSF. Furthermore, PVL of the fourth ventricle was common, and the occurrence of single lesions in the fourth ventricle was as high as 36.2%.

Combined with our series of cases and literature review, we classified the growth patterns of PVL into single-nodular type (Figure 2), cluster-like type (Figure 3), multiple-nodular type (19, 28, 30, 39), and diffuse growth type (Figure 4). The single-nodular type was the most common (53.2%), lacked imaging characteristics, and was easily misdiagnosed. The diffuse growth type was also common (21.3%), with lesions growing mainly along the choroid plexus and ventricular wall. This growth pattern may be considered the characteristic growth pattern of PVL. The cluster-like type was rare in other tumors and was also a characteristic of PVL. Multiple nodules were often considered as metastatic tumors, and this growth pattern was mostly due to the metastasis of tumor cells to other ventricles *via* CSF. Brozovich et al. reviewed a group of primary fourth ventricle lymphomas and reported that 50% of patients exhibited metastasis to other ventricles, which further confirmed the spread of PVL *via* CSF (34). Based on these data, we suggest that the presence of multiple lesions in the ventricle, especially those with cluster-like or diffuse growth along the choroid plexus and ventricular wall, is highly likely to be PVL.

### Diagnosis and Differential Diagnosis

Primary ventricular lymphomas in the lateral ventricle are more likely to be misdiagnosed as meningioma, choroid plexus papilloma, or glioma. In contrast, in the fourth ventricle, PVL is more likely to be misdiagnosed as medulloblastoma (in children), ependymoma, or astrocytoma. Multiple lesions are easily misdiagnosed as metastatic tumors or subependymal giant cell astrocytoma (SEGA). The imaging characteristic for diagnosis of PVL, in addition to characteristic growth patterns on MRI examination, is hypointensity on T1- and T2-weighted imaging; contrast enhancement typically shows homogeneous enhancement, with modest surrounding edema, and diffusion-weighted imaging usually shows uniform restricted diffusion (44). Multifocality of meningioma is rare, and choroid plexus papilloma usually presents a cystic, isointense T1-weighted image, and both have a clear boundary and slowly progression (45, 46); medulloblastoma and glioma are typically have high signal on T2-weighted imaging, and contrast enhancement tends to be heterogeneous (47); metastases with a pure intraventricular location are rare, usually with severe brain edema, and SEGA, which commonly occurs in younger patients, is almost always located in the vicinity of the foramen of Monro, with high-density calcification on CT examination (48). CSF is widely used in the diagnosis of PCNSL, including conventional cytology analysis, flow cytometry, polymerase chain reaction, detection of monoclonal B cells, and the recently reported detection of microRNA and interleukin-10 levels, which facilitate the diagnosis of PCNSL (49). However, only 15% of patients with PCNSL with CSF involvement are detectable by CSF examination (4). Patients with PVL may have a higher risk of spread *via* CSF. As a low-risk method, the value of this approach needs further investigation.

Because of the rarity of PVL, noninvasive diagnosis is difficult, and all eight patients in this group were misdiagnosed or could not be diagnosed before surgery. Therefore, obtaining pathological examination through biopsy or surgery is still a necessary means for diagnosing PVL. After a definite diagnosis of intracranial lesions, systemic examinations, including thoracic and abdominal bone marrow punctures, are also necessary for excluding systemic lymphomas involving the central nervous system (50).

### Treatment

Considering the rarity of PVL in clinical practice, there is a lack of systematic experience in the treatment of PVL, and treatment is mostly performed according to the principles of PCNSL, including chemotherapy, radiation, and immunotherapy. HDX-based induction chemotherapy is currently the first-line therapy (51). Whole-brain radiotherapy for PCNSL remains controversial (52). As PCNSL has an infiltrative growth pattern, some lesions are multiple and deep, making surgery difficult. Studies have demonstrated that surgical resection did not significantly prolong survival time in patients (53). As a result, surgical resection was not included in the standard treatment plan and was limited to biopsy. With the recent progress in surgical techniques, such as the innovative use of fluorescein sodium, safe resection has been increasingly performed in patients with PCNSL (54). Furthermore, studies have demonstrated that, compared with biopsy, GTR and STR significantly prolong progression-free survival and overall survival time in patients with PCNSL (55). Some researchers propose that, for PCNSL with solitary nodules, superficial position, or acute cerebral hernia, surgical resection should be incorporated into treatment strategies (50). In this study, 26 (55.3%) of 47 patients underwent surgical resection and 50% (13/26) achieved GTR. Only one patient who underwent surgery in our center developed respiratory failure after surgery, and no other patients experienced serious complications; thus, surgery is safe. PVL in the third and fourth ventricles, lesions that easily blocked the CSF circulation pathway and led to obstructive hydrocephalus, and ventriculoperitoneal shunt increased the risk of tumor cell implantation metastasis (56). Surgery not only can gain tumor tissue for pathological diagnosis but also can discharge obstructive hydrocephalus. Hence, for solitary PVL, especially in patients with acute obstructive hydrocephalus, surgical resection is a feasible treatment. For cluster-like lesions that can be completely resected, surgical resection may also be considered. Given the established and wide application of endoscopic technology, endoscopic resection can be considered, as reported in the literature (37). A biopsy is recommended as the first choice for patients with diffuse growth and multinodular growth. In patients with partial lesions invading the medial thalamus, brainstem, and other critical functional areas, surgery should be considered with caution.

Studies have confirmed that prognosis is poorer in patients with PCNSL treated with surgical resection alone than in those treated with postoperative radiotherapy and chemotherapy, with a median survival time of only 4.6 months (57, 58). Among our eight patients, five did not receive any postoperative adjuvant therapy, and all of them relapsed and died, except for one patient who discontinued treatment due to complications, the median survival time was only 5.5 months. Radiotherapy and chemotherapy were considered essential for the treatment of PVL.

### Limitations

The sample size of this study was small, and the literature was mainly based on case reports. The study lacked a large sample size and detailed follow-up. In particular, only the survival status of patients was followed up in the literature, and the follow-up time was too short to analyze the factors influencing patient prognosis.

## Conclusion

Primary ventricular lymphoma is a rare disease that manifests clinically as increased intracranial pressure, but this symptom is nonspecific. Because of noninvasive diagnosis, the condition is commonly misdiagnosed. Cluster-like growth along the choroid plexus and diffuse growth along the ventricular wall constitute key imaging characteristics. For localized lesions, especially in patients with acute obstructive hydrocephalus, surgical resection is feasible, and postoperative adjuvant radiation and chemotherapy are necessary.

## Data Availability Statement

The original contributions presented in the study are included in the article/Supplementary Material, further inquiries can be directed to the corresponding author/s.

## Ethics Statement

The studies involving human participants were reviewed and approved by Ethics Committee of Tongji Hospital affiliated with Tongji Medical College of Huazhong University of Science and Technology. Written informed consent for participation was not required for this study in accordance with the national legislation and the institutional requirements.

## Author Contributions

LC and KS concepted and designed the study. HZ, JiW, GW, and XM contributed to acquisition of data. KZ and JuW contributed to analysis and interpretation of data. LC drafted the article. KS approved the final version of the manuscript on behalf of all authors and contributed to study supervision. All authors critically revised the article and reviewed submitted version of the manuscript.

## Funding

This work was supported by the Natural Science Foundation of Hubei Province (Grant No. 2020CFB657).

## Conflict of Interest

The authors declare that the research was conducted in the absence of any commercial or financial relationships that could be construed as a potential conflict of interest.

## Publisher's Note

All claims expressed in this article are solely those of the authors and do not necessarily represent those of their affiliated organizations, or those of the publisher, the editors and the reviewers. Any product that may be evaluated in this article, or claim that may be made by its manufacturer, is not guaranteed or endorsed by the publisher.

## References

[1] Villano JL Koshy M Shaikh H Dolecek TA McCarthy BJ. Age, gender, and racial differences in incidence and survival in primary CNS lymphoma. Br J Cancer. (2011) 105:1414–8. 10.1038/bjc.2011.357 21915121 PMC3241537

[2] Dolecek TA Propp JM Stroup NE Kruchko C. Cbtrus statistical report: primary brain and central nervous system tumors diagnosed in the United States in 2005–2009. Neuro Oncol. (2012) 14:v1–49. 10.1093/neuonc/nos218 23095881 PMC3480240

[3] Rubenstein J Ferreri AJ Pittaluga S. Primary lymphoma of the central nervous system: epidemiology, pathology and current approaches to diagnosis, prognosis and treatment. Leuk Lymphoma. (2008) 49:43–51. 10.1080/10428190802311441 18821432 PMC4110179

[4] Korfel A Schlegel U. Diagnosis and treatment of primary cns lymphoma. Nat Rev Neurol. (2013) 9:317–27. 10.1038/nrneurol.2013.83 23670107

[5] Moher D Liberati A Tetzlaff J Altman DG The PRISMA Group (2009). Preferred reporting items for systematic reviews and meta-analyses: the PRISMA statement. PLoS Med. (2009) 6:e1000097. 10.1371/journal.pmed.1000097 19621072 PMC2707599

[6] Werneck LC Hatschbach Z Mora AH Novak EM. [Meningitis caused by primary lymphoma of the central nervous system. report of a case]. Arq Neuropsiquiatr. (1977) 35:366–72. 10.1590/s0004-282x1977000400010 588092

[7] Bogdahn U Bogdahn S Mertens HG Dommasch D Wodarz R Wunsch PH. Primary non-hodgkin's lymphomas of the Cns. Acta Neurol Scand. (1986) 73:602–14. 10.1111/j.1600-0404.1986.tb04607.x 3751501

[8] Haegelen C Riffaud L Bernard M Morandi X. Primary isolated lymphoma of the fourth ventricle: case report. J Neurooncol. (2001) 51:129–31. 10.1023/a:1010790325692 11386409

[9] Pascual JM Gonzalez-Llanos F Roda JM. Primary hypothalamic-third ventricle lymphoma. case report and review of the literature. Neurocirugia (Astur). (2002) 13:305–10. 10.1016/s1130-1473(02)70605-2 12355653

[10] Kelley TW Prayson RA Barnett GH Stevens GH Cook JR Hsi ED. Extranodal marginal zone b-cell lymphoma of mucosa-associated lymphoid tissue arising in the lateral ventricle. Leuk Lymphoma. (2005) 46:1423–7. 10.1080/10428190500205895 16194887

[11] Park SW Yoon SH Cho KG. An endoscopically proven ventriculitis-type, cyst-like intraventricular primary lymphoma of the central nervous system. Acta Neurochir (Wien). (2006) 148:981–4. 10.1007/s00701-006-0797-2 16791437

[12] Jung TY Jung S Lee MC Lee KH. Extranodal marginal zone b-cell lymphoma mimicking meningioma in lateral ventricle: a case report and possible pathogenesis. J Neurooncol. (2006) 80:63–7. 10.1007/s11060-006-9153-x 16628474

[13] Terasaki M Abe T Tajima Y Fukushima S Hirohata M Shigemori M. Primary choroid plexus t-cell lymphoma and multiple aneurysms in the Cns. Leuk Lymphoma. (2006) 47:1680–2. 10.1080/10428190600612503 16966285

[14] Cecchi PC Billio A Colombetti V Rizzo P Ricci UM Schwarz A. Primary high-grade b-cell lymphoma of the choroid plexus. Clin Neurol Neurosurg. (2008) 110:75–9. 10.1016/j.clineuro.2007.08.019 17928135

[15] Hill CS Khan AF Bloom S McCartney S Choi D. A rare case of vomiting: fourth ventricular b-cell lymphoma. J Neurooncol. (2009) 93:261–2. 10.1007/s11060-008-9765-4 19093074

[16] Gu Y Hou YY Zhang XB Hu F. Primary central nervous system burkitt lymphoma as concomitant lesions in the third and the left ventricles: a case study and literature review. J Neurooncol. (2010) 99:277–81. 10.1007/s11060-010-0122-z 20146089

[17] Sasani M Bayhan M Sasani H Kaner T Oktenoglu T Cakiroglu G. Primary central nervous system lymphoma presenting as a pure third ventricular lesion: a case report. J Med Case Rep. (2011) 5:213. 10.1186/1752-1947-5-213 21619666 PMC3121681

[18] Jiang M Zhu J Guan YS Zou LQ. Primary central nervous system burkitt lymphoma with non-immunoglobulin heavy chain translocation in right ventricle: case report. Pediatr Hematol Oncol. (2011) 28:454–8. 10.3109/08880018.2011.566599 21615246 PMC3157034

[19] Brar R Prasad A Sharma T Vermani N. Multifocal lateral and fourth ventricular b-cell primary cns lymphoma. Clin Neurol Neurosurg. (2012) 114:281–3. 10.1016/j.clineuro.2011.10.020 22100106

[20] Yakupoglu H Onal MB Civelek E Kircelli A Aygun MS Aygun FM. Primary diffuse choroid plexus t-cell lymphoma: case report. J Neuroradiol. (2012) 39:116–8. 10.1016/j.neurad.2011.03.002 21641645

[21] Rao RN Mishra D Agrawal P Kumar R. Primary B-cell central nervous system lymphoma involving fourth ventricle: a rare case report with review of literature. Neurol India. (2013) 61:450–3. 10.4103/0028-3886.117608 24005755

[22] Bokhari R Ghanem A Alahwal M Baeesa S. Primary isolated lymphoma of the fourth ventricle in an immunocompetent patient. Case Rep Oncol Med. (2013) 2013:614658. 10.1155/2013/614658 23607015 PMC3625557

[23] Liao CH Lin SC Hung SC Hsu SP Ho DM Shih YH. Primary large b-cell lymphoma of the fourth ventricle. J Clin Neurosci. (2014) 21:180–3. 10.1016/j.jocn.2013.02.036 24012385

[24] Fabiano AJ Syriac S Fenstermaker RA Qiu J. Primary fourth ventricular b-cell lymphoma in an immunocompetent patient. Clin Neuropathol. (2014) 33:94–7. 10.5414/NP300658 23924755 PMC4199190

[25] Grossman R Nossek E Shimony N Raz M Ram Z. Intraoperative 5-aminolevulinic acid-induced fluorescence in primary central nervous system lymphoma. J Neurosurg. (2014) 120:67–9. 10.3171/2013.9.JNS131076 24138204

[26] Alabdulsalam A Zaidi SZ Tailor I Orz Y Al-Dandan S. Primary burkitt lymphoma of the fourth ventricle in an immunocompetent young patient. Case Rep Pathol. (2014) 2014:630954. 10.1155/2014/630954 25254131 PMC4164299

[27] Funaro K Bailey KC Aguila S Agosti SJ Vaillancourt C. A case of intraventricular primary central nervous system lymphoma. J Radiol Case Rep. (2014) 8:1–7. 10.3941/jrcr.v8i3.1361 24967022 PMC4035363

[28] Zhu Y Ye K Zhan R Tong Y. Multifocal lateral and fourth ventricular primary central nervous system lymphoma: case report and literature review. Turk Neurosurg. (2015) 25:493–5. 10.5137/1019-5149.JTN.10496-14.1 26037194

[29] Hsu HI Lai PH Tseng HH Hsu SS. Primary solitary lymphoma of the fourth ventricle. Int J Surg Case Rep. (2015) 14:23–5. 10.1016/j.ijscr.2015.07.006 26209757 PMC4573413

[30] Suri V Mittapalli V Kulshrestha M Premlani K Sogani SK Suri K. Primary intraventricular central nervous system lymphoma in an immunocompetent patient. J Pediatr Neurosci. (2015) 10:393–5. 10.4103/1817-1745.174433 26962354 PMC4770660

[31] Cellina M Fetoni V Baron P Orsi M Oliva G. Unusual primary central nervous system lymphoma location involving the fourth ventricle and hypothalamus. Neuroradiol J. (2015) 28:120–5. 10.1177/1971400915576671 25923685 PMC4757149

[32] Qin JZ Wu YK Yang ZJ Lv J Dang YY Zhang HT. Endoscopic biopsy of a b-cell lymphoma involving the entire ventricular system: a case report. Exp Ther Med. (2016) 11:325–7. 10.3892/etm.2015.2861 26889262 PMC4726988

[33] Liu H Hou H Cheng J. Primary burkitt lymphoma of the fourth ventricle mimicking a medulloblastoma in a child. J Neurooncol. (2016) 127:205–7. 10.1007/s11060-015-2023-7 26703786

[34] Brozovich A Ewing D Burns E Hatcher C Acosta G Khan U. Primary Cns lymphoma arising from the 4(th) ventricle: a case report and review of the literature. Case Rep Oncol Med. (2019) 2019:2671794. 10.1155/2019/2671794 31093392 PMC6481150

[35] Wang D Su M Xiao J. A rare case of primary ventricular lymphoma presented on Fdg Pet/Ct. Clin Nucl Med. (2020) 45:156–8. 10.1097/RLU.0000000000002876 31833937

[36] Haddad R Alkubaisi A Al Bozom I Haider A Belkhair S. Solitary primary central nervous system lymphoma mimicking third ventricular colloid cyst-case report and review of literature. World Neurosurg. (2019) 123:286–94. 10.1016/j.wneu.2018.12.026 30579027

[37] Guo R Zhang X Niu C Xi Y Yin H Lin H. Primary central nervous system small lymphocytic lymphoma in the bilateral ventricles: two case reports. BMC Neurol. (2019) 19:200. 10.1186/s12883-019-1430-3 31426757 PMC6699128

[38] Ball MK Morris JM Wood AJ Meyer FB Kaszuba MC Raghunathan A. Ventricle-predominant primary cns lymphomas: clinical, radiological and pathological evaluation of five cases and review of the literature. Brain Tumor Pathol. (2020) 37:22–30. 10.1007/s10014-019-00354-x 31630277

[39] Khanna G Ahlawat S Garg N Gupta R Patir R. A rare case of isolated intraventricular primary central nervous system lymphoma in an 85-year-old man. Asian J Neurosurg. (2021) 16:623–5. 10.4103/ajns.AJNS_551_20 34660383 PMC8477809

[40] Correia CE Schaff LR Grommes C. Central nervous system lymphoma: approach to diagnosis and treatment. Cancer J. (2020) 26:241–52. 10.1097/PPO.0000000000000449 32496457

[41] Mendez JS Ostrom QT Gittleman H Kruchko C DeAngelis LM Barnholtz-Sloan JS. The elderly left behind-changes in survival trends of primary central nervous system lymphoma over the past 4 decades. Neuro Oncol. (2018) 20:687–94. 10.1093/neuonc/nox187 29036697 PMC5892148

[42] Bataille B Delwail V Menet E Vandermarcq P Ingrand P Wager M. Primary intracerebral malignant lymphoma: report of 248 cases. J Neurosurg. (2000) 92:261–6. 10.3171/jns.2000.92.2.0261 10659013

[43] Fine HA Mayer RJ. Primary central nervous system lymphoma. Ann Intern Med. (1993) 119:1093–104. 10.7326/0003-4819-119-11-199312010-00007 8239229

[44] Cheng G Zhang J. Imaging features (ct, mri, mrs, and pet/ct) of primary central nervous system lymphoma in immunocompetent patients. Neurol Sci. (2019) 40:535–42. 10.1007/s10072-018-3669-7 30580380 PMC6433804

[45] Teng H Liu Z Yan O He W Jie D Qie Y. Lateral ventricular meningiomas: clinical features, radiological findings and long-term outcomes. Cancer Manag Res. (2021) 13:6089–99. 10.2147/CMAR.S320651 34377027 PMC8349535

[46] Lin H Leng X Qin CH Du YX Wang WS Qiu SJ. Choroid plexus tumours on mri: similarities and distinctions in different grades. Cancer Imaging. (2019) 19:17. 10.1186/s40644-019-0200-1 30894223 PMC6427869

[47] Citterio G Reni M Gatta G Ferreri AJM. Primary central nervous system lymphoma. Crit Rev Oncol Hematol. (2017) 113:97–110. 10.1016/j.critrevonc.2017.03.019 28427529

[48] Beaumont TL Limbrick DD Smyth MD. Advances in the management of subependymal giant cell astrocytoma. Childs Nerv Syst. (2012) 28:963–8. 10.1007/s00381-012-1785-x 22562196

[49] Schroers R Baraniskin A Heute C Vorgerd M Brunn A Kuhnhenn J. Diagnosis of leptomeningeal disease in diffuse large b-cell lymphomas of the central nervous system by flow cytometry and cytopathology. Eur J Haematol. (2010) 85:520–8. 10.1111/j.1600-0609.2010.01516.x 20727005

[50] Hoang-Xuan K Bessell E Bromberg J Hottinger AF Preusser M Ruda R. Diagnosis and treatment of primary cns lymphoma in immunocompetent patients: guidelines from the european association for neuro-oncology. Lancet Oncol. (2015) 16:e322–32. 10.1016/S1470-2045(15)00076-5 26149884

[51] Yang H Xun Y Yang A Liu F You H. Advances and challenges in the treatment of primary central nervous system lymphoma. J Cell Physiol. (2020) 235:9143–65. 10.1002/jcp.29790 32420657

[52] Ferreri AJ DeAngelis L Illerhaus G O'Neill BP Reni M Soussain C. Whole-brain radiotherapy in primary cns lymphoma. Lancet Oncol. (2011) 12:118–9; author reply 9–20. 10.1016/S1470-2045(11)70018-3 21277546

[53] Schellekes N Barbotti A Abramov Y Sitt R Di Meco F Ram Z. Resection of primary central nervous system lymphoma: impact of patient selection on overall survival. J Neurosurg. (2021) 135:1016–25. 10.3171/2020.9.Jns201980 33636699

[54] Schebesch KM Hoehne J Hohenberger C Acerbi F Broggi M Proescholdt M. Fluorescein sodium-guided surgery in cerebral lymphoma. Clin Neurol Neurosurg. (2015) 139:125–8. 10.1016/j.clineuro.2015.09.015 26432995

[55] Weller M Martus P Roth P Thiel E Korfel A German PSG. Surgery for primary cns lymphoma? challenging a paradigm. Neuro Oncol. (2012) 14:1481–4. 10.1093/neuonc/nos159 22984018 PMC3499010

[56] Chen F Guo H Mao C Jiang X Liu S Huang L. Unusual relapse of primary central nervous system lymphoma both inside and outside central nervous system in patient with ventriculoperitoneal shunt. World Neurosurg. (2019) 127:625–8. 10.1016/j.wneu.2019.01.277 30794971

[57] Wang H Wang M Wei J Wang L Mao L Jin J. Primary central nervous system lymphoma: retrospective analysis of 34 cases in a single centre. J Int Med Res. (2018) 46:883–94. 10.1177/0300060517734395 28984175 PMC5971520

[58] Henry JM Heffner RR Dillard SH Earle KM Davis RL. Primary malignant lymphomas of the central nervous system. Cancer. (1974) 34:1293–302. 4607602 10.1002/1097-0142(197410)34:4<1293::aid-cncr2820340441>3.0.co;2-p

