# Supplementary material for: Clinical Features, Diagnosis, and Treatment of Primary Intraventricular Lymphoma: Insights From a Monocentric Case Series
Source: Front Neurol. 2022 Jun 6;13:920505. doi: 10.3389/fneur.2022.920505 (PMC9207404; doi:10.3389/fneur.2022.920505)
Supplement: Supplementary file 1 [file Data_Sheet_1.docx]

**Supplementary Table 1 Summary of 39 cases from previously published case series**

| Authors & Year | Age (yrs),  Sex | Clinical  Presentation | Location | Growth Pattern | Extent of  Resection | Adjuvant Therapy | Pathology | FU（mos),  Status |
| --- | --- | --- | --- | --- | --- | --- | --- | --- |
| Werneck et al., 1977^5^ | 17, F | Meningitis | 4th V | NR | ND | ND | PCNSL^＃^ | Died |
| Bogdahn et al., 1986^6^ | 51, M | Seizures | LV | NR | BP | CMT, RT | HG-TCL | 16, Died |
| Haegelen et al., 2001^7^ | 33, F | Headache, vertigo | 4th V | Solitary nodular | SPR | CMT, RT | HG-BCL | 7,SA |
| Pascual et al., 2002^8^ | 57, F | Decreased vision, confusion | 3rd V | Solitary nodular | SPR | CMT, RT | BCL | 6, Alive |
| Kelley et al., 2005^9^ | 53, M | Headache, seizures | 3rd V | Solitary nodular | GTR | CMT | BCL | 14, Alive |
| Park et al., 2006^10^ | 55, F | Memory deficit | LV | Diffuse type | BP | RT | BCL | NR |
| Jung et al., 2006^11^ | 63, M | Confusion, monoparesis | 3rd V | Solitary nodular | GTR | CMT | BCL | NR |
| Terasaki et al., 2006^12^ | 56, M | Headache | L V | Solitary nodular | GTR | CMT, RT | TCL | 19, Alive |
| Cecchi et al., 2008^13^ | 71, F | Speech disturbance,  hemiparesis | 3rd V | Solitary nodular | GTR | CMT, RT | HG-BCL | 26, Alive |
| Hill et al., 2009^14^ | 69, M | Vomiting, nausea | 4th V | Solitary nodular | BP | CMT | HG-BCL | 3, Alive |
| Gu et al., 2010^15^ | 75, M | Headache, speech disturbance | LV, 3rd V | Solitary nodular | GTR | RT | PCNSBL | 9, Alive |
| Sasani et al., 2011^16^ | 37, M | Headache | 3rd V | Solitary nodular | GTR | ND | PCNSL | 12, Alive |
| Jiang et al., 2011^17^ | 14, M | Headache | LV | Solitary nodular | GTR | CMT, RT | PCNSBL | 18, Alive |
| Brar et al., 2012^18^ | 65, F | Headache,vomiting, nausea | LV, 4th V | Multiple nodular | BP | CMT | HG-BCL | 2, Alive |
| Yakupoglu et al., 2012^19^ | 63, F | Seizure | EVS | Diffuse type | BP | ND | TCL | NR |
| Rao et al., 2013^20^ | 59, M | Vomiting, nausea,  vertigo | 4th V | Solitary nodular | SPR | CMT | DLBCL | 8, Alive |
| Bokhari et al., 2013^21^ | 50, M | Vomiting, nausea | 4th V | Solitary nodular | GTR | CMT, RT | HG-BCL | 18, Alive |
| Liao et al,2014^22^ | 77, M | Vomiting, unsteady gait, vertigo, nausea | 4th V | Cluster like | GTR | ND | DLBCL | 9, Alive |
| Fabiano et al., 2014^23^ | 60, F | Diplopia | 4th V | Solitary nodular | SPR | CMT, RT | DLBCL | 6, Alive |
| Grossman et al., 2014^24^ | 66, M | Ataxia,diplopia | 4th V | Solitary nodular | SPR | ND | PCNSL | NR |
| Alabdulsalam et al., 2014^25^ | 18, M | Ataxia, cranial nerves palsies | 4th V | Solitary nodular | GTR | CMT | PCNSBL | 18, Alive |
| Funaro et al., 2014^26^ | 68, M | Headache, unsteady gait | EVS | Diffuse type | BP | ND | DLBCL | Died |
| Zhu et al., 2014^27^ | 66, M | Dizziness, diplopia | LV,  4 th V | Multiple nodular | BP | CMT | DLBCL | 6, Alive |
| Hsu et al., 2015^28^ | 61, M | Headache, dizziness,  ataxia | 4 th V | Solitary nodular | SPR | CMT | DLBCL | 3, Alive |
| Suri et al., 2015^29^ | 15, M | Headache, vomiting, nausea | LV, 4th V | Multiple nodular | BP | ND | DLBCL | NR |
| Cellina et al., 2015^30^ | 65, M | Headache, vomiting, nausea | EVS | Diffuse type | BP | IMT | DLBCL | 2, Alive |
| QIN et al , 2016^31^ | 62, M | Vomiting, nausea | EVS | Cluster like | BP | CMT, RT | DLBCL | 7, Alive |
| Liu et al., 2016^32^ | 6, M | Headache | 4th V | Solitary nodular | SPR | CMT | PCNBL | 6, Alive |
| Brozovich et al., 2019^33^ | 65, M | Vomiting, nausea | 4th V | Solitary nodular | BP | CMT, RT | DLBCL | 8, Alive |
| Wang et al., 2019^34^ | 51, F | Unsteady gait , memory deficit | EVS,SC | Diffuse type | BP | ND | DLBCL | NR |
| Haddad et al., 2019^35^ | 72, M | Confusion, memory deficit | 3rd V | Solitary nodular | SPR | CMT | BCL | 11, Alive |
| Guo et al., 2019^36^ | 45, F | Headache | LV | Diffuse type | BP | CMT | SLL | 12, Alive |
|  | 49, M | Headache, dizziness | LV | Diffuse type | BP | CMT | SLL | 12, Alive |
| Ball et al., 2019^37^ | 75, F | Memory deficit | LV | Diffuse type | BP | RT | DLBCL | 4, Died |
|  | 60, M | Headache, fatigue | 4th V | NR | BP | ND | DLBCL | 1, Died |
|  | 74, M | Ataxia, diplopia | EVS | Diffuse type | BP | ND | DLBCL | 1.6, Died |
|  | 40, M | Headache, diplopia | 3rd V | Solitary nodular | SPR | CMT | DLBCL | 9, Alive |
|  | 64, F | Nausea, dizziness | EVS | Multiple nodular | BP | CMT | DLBCL | 14, Alive |
| Khanna et al., 2019^38^ | 85, M | Vision loss,  memory deficit | LV, 3rd V | Multiple nodular | BP | ND | DLBCL | NR |

EVS = entire ventricles system, SC = spinal cord, BP = biopsy, SPR = subtotal or partical resection, IMT = immunotherapy, HG = high grade, TCL = T-cell lymphoma, BCL = B-Cell lymphoma, DLBCL = diffuse large B-cell lymphoma, NHL = Non-Hodgkin lymphoma, PCNSBL = primary central nervous system Burkitt lymphoma, NR = not registered, SLL = small lymphocytic lymphoma, #diagnosis was confirmed in the post-mortem examination, ASCT = auto logousstem cell transplantation.

**Supplementary Table 2 Summary of clinical. characteristics of the case series and review**

| Characteristics | N or Mean（SD） | N(%) |
| --- | --- | --- |
| **Female** | 13 | 27.7 |
| **Age, years (range)** | 55±18(6-85) |  |
| **Presenting symptoms** |  |  |
| Headache, Vomiting, nausea | 28 | 59.6 |
| Paresis, Ataxia, unsteady gait | 12 | 25.5 |
| Dizziness or vertigo | 11 | 23.4 |
| Diplopia | 6 | 12.8 |
| Deterioration of memory | 6 | 12.8 |
| Other symptoms | 8 | 17.0 |
| **Imaging features** |  |  |
| Solitary | 28 | 59.6 |
| Multiple | 19 | 40.4 |
| **Location** |  |  |
| Lateral ventricles | 9 | 19.1 |
| Third ventricles | 7 | 14.9 |
| Fourth ventricles | 17 | 36.2 |
| Multiple ventricles  and spinal cord | 14 | 29.8 |
| **Growth pattern** |  |  |
| Solitary Nodular | 25 | 53.2 |
| Diffuse Type | 10 | 21.3 |
| Multiple Nodular | 5 | 10.6 |
| Cluster-Like | 4 | 8.5 |
| Not registered | 3 | 6.4 |
| **Operation or Examination** |  |  |
| Gross total resection | 12 | 25.6 |
| Partial resection | 14 | 29.8 |
| Biopsy | 19 | 40.4 |
| CSF examination | 1 | 2.1 |
| Post-mortem examination | 1 | 2.1 |
| **Pathology** |  |  |
| BCL | 35 | 74.5 |
| Burkitt | 4 | 8.5 |
| TCL | 3 | 6.4 |
| SLL | 2 | 4.3 |
| PCNSL | 3 | 6.4 |
| **Adjuvant therapy** |  |  |
| Chemotherapy only | 14 | 29.8 |
| Radiotherapy only | 3 | 6.4 |
| Chemotherapy + Radiotherapy | 13 | 27.7 |
| Immunotherapy | 1 | 2.1 |
| No Adjuvant therapy | 16 | 34.0 |
| **Outcome** |  |  |
| Died, ST (Months)（Range） | 12, 7.0 ± 7.2 M**^＃^** (1-18) | 25.5 |
| Still alive, FU (Months)（Range） | 28, 12.4 ± 10.3M (0.5-48) | 61.7 |
| Not registered | 7 | 12.8 |

**＃**The survival time of three patients who died in the hospital was not included (References 5, 26, and our case No.7)
